# The Law, Dementia, and Sexuality—Is the Law Striking the Right Balance?

**DOI:** 10.1093/geront/gnae112

**Published:** 2024-08-14

**Authors:** Oluwatoyin Sorinmade, Alex Ruck Keene, Carmelle Peisah

**Affiliations:** Department of Old Age Psychiatry, Kent and Medway NHS and Social Care Partnership Trust, Maidstone, UK; Dickson Poon School of Law, King’s College London, London, UK; Barrister, 39 Essex Chambers, London, England, UK; School of Clinical Medicine, University of New South Wales, Sydney, New South Wales, Australia; Faculty of Medicine and Health, University of Sydney, Sydney, New South Wales, Australia

**Keywords:** Ethics, Person-centered care, Quality of care

## Abstract

**Background and Objectives:**

English and Welsh laws require “contemporaneous” consent to sexual relations, often precluding “non-capacituos” people living with advanced dementia from these human interactions.

**Research Design and Methods:**

The views of people living with dementia, carers, professionals, and over-55s were explored on implications of current laws on sexuality in dementia. Thirty-five participants from England were recruited through purposive selective sampling. Audio-taped semistructured interviews were transcribed and thematically analyzed with each stakeholder group coded separately.

**Results:**

Common themes across stakeholders were (i) law reform needed due to their hammer-like effect ignoring the individual; (ii) dissonant theme of needing the law for protection; (iii) negative impact of denied intimacy on individuals and partners; (iv) support for Advance Decisions on Intimacy with caveats; (v) less support for involvement of Court of Protection and Powers of Attorney; (vi) call for review of capacity concept with support for an assent model. People living with dementia described shame and stigma associated with policing of their sexuality and perception of being singled out for protection and intrusion into their lives. For informal carers (mostly wives), the theme of “what about me” emerged, demonstrating that for the long-term partnered, this is a couple’s issue.

**Discussion and Implications:**

Practical implications of this study include need to address ageism and ableism; human rights education for society and professionals; starting point of presumed capacity for sexual relations with consensus on how and when this should be rebutted; and care homes’ certification requirements should incorporate assessment of the relationship health of their residents.

For very many, the need for intimacy and expression of sexuality exists, regardless of age or disability. Importantly, the presence of dementia does not erase sexuality, and physical tenderness and intimacy are intrinsic to the well-being of many older persons living with dementia, rendering it an important part of active aging with dementia (Eshmaway, 2022; Freak-Poli et al., 2018; Granville et al., 2018; Lindau et al., 2018). At the same time, there is huge variability in sexual expression among individuals living with dementia. Although many people living with dementia remain interested in sexual pleasure and are sexually active well into the disease, there are others who (gradually) experience sexual indifference (Dalmer & Marshall, 2023; Granville et al., 2018; Lindau et al., 2018). For others, the disease process can still lead to poor impulse control and impaired reasoning manifesting as sexually inappropriate behavior (D’cruz, Andrade & Rao, 2020; De Giorgi & Series, 2016).

As is the case with individuals without dementia, different factors influence intimacy needs and expression of sexuality in persons living with dementia, including their premorbid personality, the quality and nature of their interpersonal relationships, their gender identity, sexual identity, and premorbid intimacy and sexual expression, as well as their physical health and level of functioning and that of their partner (Frauke &Enzlin, 2023; Mahieu and Gastmas, 2015). Further variability is conferred by both dementia subtype and severity of disease. For example, some people with frontotemporal dementia might become less inhibited and display intimacy-seeking behavior more often, in an aggressive way and in some instances out of character for them (Chapman & Spitznagel, 2019), and conversely, they might become apathetic and less interested in sexual relations.

Clearly, there is no one-size-fits-all approach to sexuality and dementia, except when it comes to the law. Many of our laws, as they stand, prescribe a single solution that errs on the side of protection. The laws in England and Wales are clear on the need for “here and now” consent to engage in sexual relations (Mental Capacity Act, 2005; Sexual Offences Act, 2003). Although such laws purposefully safeguard against abuse and exploitation, they inadvertently enforce celibacy. With these laws, it becomes unlawful for persons with dementia to engage in sexual relations once they lose the “here and now” ability to give consent to such (Mental Capacity Act, 2005; Sexual Offences Act, 2003). Although it is beyond the scope of this paper to review international legislation in this area, suffice to say that such laws are echoed in several jurisdictions (Barry & Parson, 2023; O’Neill & Peisah, 2021). For the purpose of this research, we focus here on the laws in England and Wales, although they have relevance internationally.

The consequence of these laws is that persons with dementia, who have lost “here and now” ability to give consent to sexual relations, especially those in care homes, are dissuaded from engaging in such relations, and their partners criminalized for doing so. For some time, spouses have been charged with sexual assault offenses on account of engaging (or “suspected of engaging”) in intimate acts with their spouses living with dementia (Carter, 2015; Holmquist, 2015; Lindsay, 2015). The balance is further tipped toward protection by family members who feel the need to monitor and police expression of sexuality in care home residents (Bauer, Nay et al., 2014). Care home staff often struggle with finding the balance between supporting residents’ rights to make decisions about, and enjoy intimacy, and their duty of care and compliance with legislation (Dalmer &Marshall, 2023; Director, 2023; Rector et al., 2020; Rowntree and Zuffrey, 2015; Tarzia et al., 2012). Importantly, there have been instances where persons living with dementia have been stopped from engaging in sexual relations due to either the presumption that they have lost capacity or the inability of others to recognize their decisional capacity (A v AG, 2021; Re-SF, 2020).

The question arises: Can we empower persons living with dementia when they lose the decisional capacity for sexual relations to express and satisfy innate desires for such relations (where present), while safeguarding against abuse? These often-competing goals of promoting dignity, autonomy, and expression of will and preferences, while safeguarding against abuse, are all equally important human rights articulated in Articles 12 and 16 of the Convention of Rights of Persons with Disability (CRPD).

Answers to this question might be found in legislative reforms including (i) reform of laws in relation to substitute decision making to allow best interests decisions in relation to sexual relations to be made on behalf of non-capacitous persons living with dementia (Bartlett, 2010; Boni-Saenz, 2020); (ii) review of the criteria and threshold for decisional capacity for sexual relations (Arstein-Kerslake & Flynn, 2016; Bartlett, 2010); (iii) changes in laws to allow capacitous individuals to exercise precedent autonomy and make an Advance Decision on Intimacy while they retain the ability to do so (Boni-Saenz, 2016; Holmquist, 2015; Sorinmade et al., 2021).

What we do not know is what stakeholders think about this issue and these posited solutions. Importantly, in light of ageism, ableism, erotophobia, and stigma associated with dementia (Grigorovich et al., 2022), it is those who are closest to the issue that we want to hear from, rather than from the general public. We already know that a range of determinants (e.g., age, gender, educational level) influence general public attitudes toward Advance Decisions on Intimacy (ADI; Astle et al., 2022). Given the aforementioned variation in sexuality and human experience, if we want to find truly individualized, person-centered solutions, we have to take notice of those for whom the issues are concrete, not abstract (Mahieu and Gastmas, 2015).

This study aimed to qualitatively explore the views of (i) persons living with dementia, (ii) their partners, (iii) paid carers, (iv) professionals with expertise in dementia, and (v) persons over the age of 55; regarding the expression of sexuality in persons living with dementia, the impact of the current laws in England and Wales on such expression, and their views on the need for law reform and the proposition of an Advance Decision on Intimacy.

## Method

### Justification for Methodological Approach

Mindful of our goal of gaining perspectives on the laws which are largely unfamiliar, we chose the process of Deliberative Democracy using qualitative methods. Proponents for the process of Deliberative Democracy (which allows citizens to consider and reflect on policy or action that will best produce the public good) argue for public consultation to allow the community to offer informed judgments about law after being informed of such (Fishkin, 2009; Syh, 2016). Aligned with our aims, this process can facilitate the exploration of the perspectives of relevant groups—persons living with dementia, their informal and formal carers, professionals, and older persons generally—regarding the existing laws, their impact, and posited solutions. Schoenberg et al. (2011, p. 281) highlight the value of qualitative research in doing just that, namely capturing the voice of “multiple stakeholders and divergent perspectives, examining the whole context rather than its constituent parts.”

### Sampling Strategies

The study was based in England. We used purposive selective sampling of participants representative of each group to ensure a fit between sampling and research questions (Schoenberg and McAuley, 2007) and maximize the generation of information-rich data (Braun and Clarke, 2013). Convenience sampling was used by advertising the study on social media and by word of mouth, that is, approaching care home managers, support groups for persons with dementia, carers’ groups, clinic patients, and professional colleagues. Several rounds of recruitment were conducted between November 2022 and August 2023 with the goal of data saturation to determine sample size.

### Inclusion Criteria

Inclusion criteria for all participants were (i) English fluency sufficient for interview; (ii) willingness for interview to be audio-recorded for analysis; (iii) aged over 18 or over; and (iv) capacity to give informed consent. Specific group inclusion criteria were for (i) People living with dementia: diagnosis of dementia (diagnosed in clinic or with established dementia diagnosis, but living in the community); (ii) Paid Carers: current employment in a care home supporting people living with dementia with minimum of one year’s full-time experience; (iii) Unpaid Carers: being an unpaid partner or primary carer for a person living with dementia either in the community or in a care home; (iv) People aged over 55 without dementia: as stated; (v) Professionals: professionals with expertise in caring for people living with dementia.

### Data Collection

The research question lent itself to a Theoretical Thematic Analysis approach (Braun and Clarke, 2013). A semistructured interview was developed based on the gaps in the literature and the study aims to stay close to the theoretical framework (see Supplementary Appendix 1 for Interview Guide). Open-ended questions were used, and the interviewer (the First Author O. Sorinmade, an old age psychiatrist) adopted a flexible and empathic stance to accommodate the sensitivity of this topic; at the same time, using principles of Deliberative Democracy, which “offers medical ethics a promising way to consult public preferences while ensuring these are adequately informed and considered” (Kuylen et al., 2021, p. 291). Questions were prefaced by information regarding the current laws, suggestions about possible changes to current laws, including the Advance Decision on Intimacy concept. Participants had also been sent information leaflets before hand, on the current position of English and Welsh laws on mental capacity and sexual relations, as well as on the Advance Decision on Intimacy concept. The interviews were recorded and transcribed using NVivo.

### Data Analysis

All five groups were analyzed separately to capture the unique voice of each stakeholder group. Interview transcripts were initially open coded, line by line by creating memos and identifying patterns and emerging themes. This initial coding generated semantic themes to stay close to the participant voice. Subsequent coding involved grouping congruent experiences to generate categories and latent themes, iteratively revising themes using constant comparison (Braun and Clarke, 2013). The process was reliant on strong collaboration between multiple coders, that is, the three authors (Schoenberger et al., 2011). Although the first author O. Sorinmade undertook most of the coding, subsets of transcripts were independently coded by authors C. Peisah and A. Ruck Keene at all stages, with differences in coding and themes discussed prior to finalization of the higher-order codes and themes.

### Ethics

The study was approved by Wales Research Ethics Committee 2, Cardiff, Wales, and sponsored by the Kent and Medway NHS and Social Care Partnership Trust, Kent, England.

## Results

### Sample

A total of 35 participants participated in the interviews and survey. This included people living with dementia (*n* = 3), unpaid informal carers (*n* = 4), people over 55 (*n* = 6), paid carers (*n* = 8), and experts in the care of people with dementia (*n* = 14). The sample sizes for the paid carer, people over 55, and professional groups were determined by data saturation, whereas the sample sizes for people living with dementia and unpaid carer groups were determined and limited by the response to recruitment.

The demographics and characteristics of the sample are outlined in Table 1.

**Table 1.** Demographic Details of Participants

Group	*N*
People living with dementia	3
Age	Range 50–71
	Mean 62
Gender	
Female	1
Male	2
Unpaid informal carers	4
Age	Range 53–81
	Mean 72
Gender	
Female	4
Relationship	
Partner	3
Daughter	1
People over 55 years without dementia	6
Age	Range 59–71
	Mean 65
Gender	
Female	1
Male	5
Paid carers	8
Age	Range 30–55
	Mean 41
Gender	
Female	5
Male	3
Professionals	14
Gender	
Female	5
Missing data	1
Male	8
Professional background	
Barrister/solicitor	3
Psycho-geriatrician	4
Admiral nurse	1
Community psychiatric nurse	1
Dementia ward manager	1
Social worker	2
Social worker/best interests assessor	2

*Notes*: The demographic data collection tool was inclusive of gender fluidity. However, participants identified as either male or female, with one professional in dementia care, opting not to specify their gender.

### Thematic Analyses

Detailed reporting of themes derived from the analysis of each stakeholder group with illustrative quotes are presented in Tables 2–6.

**Table 2.** Themes From People Living With Dementia

Theme	Comment	Illustrative quotes
The law dehumanizing, depriving autonomy and human rights	Importance of sexuality and the perception that there is more protection and intrusion in the lives of People Living With Dementia than others.	*Sexuality [sic] it’s part and parcel of living, and I don’t think somebody should be able to make that choice for you.. it de-humanises that person.* ***(Female)*** *Depriving someone of their human rights. * ***(Male)*** *Let’s face it people are taken advantage of without saying yes or no without having dementia and things can happen unfortunately one way or the other.* ***(Male)***
The law causing shame and stigma		*It’s almost comes across as you doing something naughty. Or wrong … can bring out feelings of shame.* ***(Female)*** *Law … increasing the stigma against those people with diagnosis of dementia … and being seen as.. not capable of making any decision.* ***(Female)***
Law offering protection		*Think of situation when the person with dementia forces themselves on their intimate partner.* *Laws need to be there to prevent someone from being taken advantage of.* ***(Male)***
Consequences of thwarted sexuality	Linked to changed behaviors.	*You’re going to increase negative behaviour … because it’s a natural thing, …. people might just still display those behaviours because of, that need or desire … those around that person, might be family or partners or carers … can make a lot of strain on that relationship and a lot of problems can arise.* ***(Female)*** *frustration causes aggression. * ***(Male)***
Difficulty in assessing consent and capacity	Clues for capacity assessment (task-specific aspects, premorbid wishes, current mental state and behavior, including nonverbal signs).	*they should look at what specific thing it was that they want, when it was, where it was…. was anybody else involved, and … the behaviour of both people at that time. And the knowledge of that person and from those people that know them well. How they present on a daily basis. * ***(Female)***
People Living With Dementia should be talking about their sexuality	Needs to be part of care plan, for Safeguarding, for individualized approach.	*It may be one of those things that should be should be addressed when somebody is being given the diagnosis. And when they are followed up by different services. I think perhaps it should be included in their care plan.* ***(Female)*** *Conversations should not just be about medication or about what you’re eating or how you feel it should be all of these things collectively.* ***(Female)***
Advance Decisions on Intimacy not for everyone	Although Advance Decisions on Intimacy was not specifically discussed with People Living With Dementia, a participant spontaneously introduced the concept and its limitations (need to be individualized, inability to predict future self, dynamic nature of will and preferences).	*People don’t want to go down then they won’t. It’s not the be all and end all. In another group I have been at, everyone said that they would not want to go down that route … to sign a bit of paper to say that they give consent now as they know that they might not be able to give consent in future. It’s one of those things with dementia, you can change from minute to minute and hour to hour, your decision might change. You might consent now and say no later.* ***(Male)***
Can the law recognize assent?		*there are more ways to say yes and no than verbally saying it. …. the law should accept intent even if non-verbal.* **(Male)**

**Table 3.** Themes for Unpaid Carers

Theme	Illustrative quotes
What about me, the spouse?	*The People Living With Dementia who are becoming much more demanding than they had been in previous life in previous years and relationships change., and the person who’s the carer is finding it difficult to cope. It looks as if your view is on the People Living With Dementia How about the view on the other side of the relationship?* ***(Wife)***
Daughters very clear about what is ok	*From my own personal experience, I hope that my Father will still be allowed to kiss and cuddle my mother now that she is in a care home. I would hate for that not to happen. And that’s appropriate behaviour. I would not agree with that to be stopped because my mom wasn’t able to consent to it.* ***(Daughter)*** *My mother who has been married to my father for over 56 years and I have witnessed their relationship for as long as I’ve been alive and know that their relationship is trustworthy and reliable, and that it is not open to being abused. My mom is now in a care home, but she has only been there for a week. It will be a shame for her not to still be exposed to some degree of intimate relationship with my dad, as much as they have done leading up to this stage.* ***(Daughter)***
You can’t legislate for the myriad of individual circumstances and relationships	*Every individual case will be different. So, I don’t think you can legislate for all, there will not be one answer for everybody.* ***(Wife)***
What is consent and how much is enough?	*People might not understand the ins and out of the situation as long as they understand the salient points.* **(Daughter)**
Advance Decisions on Intimacy Pros—Giving voice	*First of all, it allows the discussion to be had. Whereas otherwise, the discussion probably wouldn’t be had. And secondly, it allows that person to be able to have a voice over the intimate aspects of their lives … should still have a voice about all aspects of their lives and intimacy is a very significant part of their lives …having said that, despite the advance decision it would be important to protect the person from harm … should be a mechanism for the advance decision to be reviewed as time goes by, to protect people from harm.* ***(Daughter)***
Advance Decisions on Intimacy Cons—May put the partner in a difficult position change of mind, need for review	*I will be willing to consent to hugging and kissing and that sort of thing, but I don’t want full penetration and so on, so if you could do that, you know, that may be more understandable, but I’m just thinking it puts the people who are actually carers or maybe their other half in a difficult position, really. I think so.* ***(Wife)*** *If it [*Advance Decisions on Intimacy*] is documented, the document might go missing. Also, the individual will not be able to change their mind though they made the document 10 years ago. They will not be able to say well I ‘ve changed my mind. And it can be abused too.****(Wife)*** *There should be a mechanism for the advance decision to be reviewed as time goes by, to protect people from harm.****(Daughter)*** *I’m not sure that anyone will really know in advance how they’re going to feel.****(Wife)***
Gender flavor Wives feel very protective of husbands but also feel obligated. Concerns regarding powerlessness of wives with overdemanding partners living with dementia, wives needing sexual respite, not feeling compelled.	*I would not know if he still wants what he wanted when he made the decision. I would feel that I am letting him and myself down.* ***(Wife)*** *if that person at a later date becomes very demanding—and if you’ve agreed earlier on that, you know you will concede to their wishes and suddenly they’re very, really demanding and your relationships change—it’s not a comfortable situation for the person who has got the capability, and you could see that happening. And I know it happens when people told me about it …* ***(Wife)*** *But I also think everyone needs comfort in their lives and probably more so when they are confused. For example, if my husband ends up going into a home, he would feel absolutely bereft of any strangers and he would probably become quite aggressive, actually, if the comfort is not there.* ***(Wife)***

**Table 4.** Themes From People Over 55 Without Dementia

Themes	Illustrative quotes
Harsh blanket laws	*there is a very good reason for the law, which is to protect vulnerable people. But it feels like a bit of a heavy-handed blanket approach … it’s not necessarily thinking about the other side that actually people might still want to have intimacy though they might not have the capacity to be able to consent.* ***(Male)*** *if people have a natural desire for something and they are being stopped and their partners are being deprived, then the law needs to be looked at.* ***(Male)*** *The fact that an individual has dementia and cannot consent to sexual relations does not mean that they do not want intimacy with their spouse. So, people who have been married for such a long-time people who are used to going for walks together, who are both retired and it’s just the two of them in the home for many years. And because one of them has now lost mental capacity due to dementia, it means the other cannot express his love for his partner. Yeah, and I think that’s wrong.* ***(Male***)
Need to safeguard against abuse	*if the person who cannot consent continues to have sexual relations for example intercourse, who would police this to ensure that they are not being made to do what they do not want to do? Would someone stand there to ensure that a husband is not making his wife, who has dementia, to do what he knows that she does not like?* ***(Male)***
Distinguishing affection from sexual intimacy	*it’s very harsh to have a blanket rule because in my eyes, sexual intercourse is very different from having a hug or kiss. I think that a hug from your loved one or a little kiss or something is acceptable but not intercourse once people stop being able to consent.* ***(Female)*** *I would imagine that the person with dementia could still receive a lot of comfort if they had a cuddle from their partner. … if not allowed that, then I think that’s taking everything away from them. People lose their dignity and a lot of things because of dementia and taking away a hug or cuddle is just another comfort taken away from them.* ***(Female)***
Fear of the future with such laws in place	*It could potentially lead me to feeling very frustrated and that my wishes, my desires, my thoughts, no matter how muddled I might be, are no longer being taken into account. It could leave me not understanding why my wife, my partner or whatever doesn’t want to have relationships with me because I might not understand that, she’s not allowed to. All I see is that she’s not. So, is that because she doesn’t like me anymore, because she’s gone off with somebody else? You know, I might start to struggle to understand why I’m not allowed to have relations with my wife … of course, you can start to think about all sorts of things.* ***(Male)*** *I would be upset and think that my husband does not care for me or love me anymore, or that he no longer wants to bother with me. On the other hand, I might not be bothered at all. It will all depend on how I am physically for that sort of thing, but it would be difficult that there would be no more cuddling and kissing. I think it would be frustrating and make me very angry. It would bring the worst out of me especially if the dementia is such that I cannot say what is bothering me. It must be sad for people that aren’t living the sort of life they’d like to lead right up to the very end, especially if they have been in a loving relationship.* ***(Female)***
Advance Decisions on Intimacy and other solutions	
Involvement of children as proxy sexual decision makers not the answer	*this is the problem. I think that their own [sic: children’s] emotion might well get in the way. Something else is the fear of the law as well. I think it could be a massive issue for people. You know, my children could turn around and say, Yeah, it’s all right, Dad could have sex with that stranger. The rest of the family might look at that very differently and might decide to sue my children for that. And that’s how it’s difficult, you know, it’s really difficult.* ***(Male)*** *I think my lot [children] would [agree on decisions], but I can imagine that there would be families where the children do not agree and this would cause a lot of problem. I think that the whole thing will have to be reviewed anyway if one goes into a care home especially if going into the care home is not with my partner.* ***(Female)***
No consensus re-role of Power of Attorney in this, potentially an adjunctive role	*[sic: re* Power of Attorney*] As far as monetary and legal things, yes, that’s fine. But as for sex or those types of things, I think it’s not acceptable.****(Male)*** *The* Power of Attorney *by their very nature, are not independent. For example, my* Power of Attorney *are my children so it means all the emotive stuff will be involved, and that might get in the way of them making objective decisions.****(Male)*** *I think that it would be a good optional add on of some sort. But there would need to be something written down for the* Power of Attorney *to follow, to protect people who leave their homes to go into a care home.****(Female)*** *There is the worry that the individual might be abused and perhaps other family members would object to what the* Power of Attorney *does.****(Female)*** *The benefit is that the people involved would have stated their wishes in writing and the* Power of Attorney *can make sure that their wishes are followed.****(Male)***
Limited role for Court of Protection	*I guess Court of Protection will be truly independent, but is that the way to go? It feels very much like a hammer to crack a nut. It seems to me like a fairly extreme solution to a basic human need. You also have to think about the time factors, you’ve got the costs too.* ***(Male)*** *I think that would be difficult for the Court of Protection as they might not know the preferences of the individual and their ruling might not be individualised and might not serve the individual well.* ***(Male)*** *I think that would be too impersonal and would just be an administrative exercise. But Court of Protection can act as back up police where there are questions about what the* Power of Attorney *is doing.****(Male)***
Advance Decisions on Intimacy—*Pros*—a moral and ethical thing and protects my human rights	*I think the former self peering into the future for the future self is not unreasonable. That’s how I look at it.* ***(Male)*** *I think it is moral as well as ethical too for someone to plan for the future. I have no problem with that at all. (* ***Male)*** *People will be able to enjoy their fundamental human right … I feel that people’s fundamental human right is being impeded. So, if my right is being impeded because the law is trying to protect the interests of the many, I am the one, and I feel that my interest is being impeded on, that is why I agree with the advance decisions on Intimacy concept.* ***(Male)***
Advance Decisions on Intimacy—*Pros*—it starts conversations	*I don’t really know that my current self can make that judgment -- but having an advance directive, I think, gives at least some kind of framework where you’re able to begin to have your thoughts and wishes, clearly recorded. If nothing else, it may help to promote or start open conversations about these things. Bearing in mind, as I said about being prude which most of us are, if it’s something that encourages those discussions, it’s got to be a good thing because we tend to be very shut up about that,* i.e., *people think that old people can’t have sex.****(Male)***
Advance Decisions on Intimacy—*Pros—*relieving the people left behind, giving them a clear idea of what you want	*I think you’ve got to do what’s right to help your family out as well, so they know what you want otherwise it might be tough for the people left behind.* *It would make things clearer to your carers how you want to be looked after apart from the normal washing and dressing. It would really help your family out.* ***(Male)*** *I think your close relatives, your other half, particularly, especially if they were really concerned as to what people might be saying. I know of a friend whose husband does not recognise her anymore and accuses her of all sorts of things. This will make it plain for people that when you were able to consent, you said what you would like to happen.* ***(Female)*** *The benefits would be that people would have a clear idea of what you would not want and what you feel you might want. It would possibly give a little more protection to the partner too against accusations of rape. … I don’t think there’s a one size fits all … don’t think there’s going to be a single legal thing that’s going to be able to solve all of this.* ***(Male)***
Advance Decisions on Intimacy—*Cons—*posing problems for partners	*It is also possible for the person that made the advance decision to become disinhibited and have a high sex drive and become sexually aggressive as you might see in some dementias and their partners might wonder what to do because of the advance decision that the person made stating what they would want. The partner might end up becoming vulnerable too.* ***(Male)***
Advance Decisions on Intimacy should be made with spouse	*The law should make it possible for a person and their partner to write something together, because you know that most acts of intimacy involves two people. So, if the partners write something together and they say this is what we would like to happen if one or the other cannot consent to sexual relations in future, then it would be good for the law to allow that sort of thing.* ***(Male)***
Religious concerns	*As a Christian, I would say limited to one’s partner but unlimited in scope, within reason.* ***(Male)***

**Table 5.** Themes for Paid Carers

Theme	Illustrative quotes
The law is too rigid with blanket laws for all	*Law is too black and white. Its just yes or no, and it’s not a yes or no answer. I don’t think when you’ve got dementia, it should depend on the dementia only and if you say yes or no. So many factors should be looked at. (* ***Female)*** *I think that it [the law] is subjective. From the point of view of basic human interactions, may be the law is not striking the right balance, but it is safeguarding people from abuse, exploitation and things like that.* ***(Male)***
Lack of understanding of the law by care staff	*we need a consistency of approach because I think the reality of it is that people on the frontline, the care home staff feel like they were the ones who have to reach these decisions and probably doing it without knowing what the law is anyway … if you question any of my staff, what is the law around it they would struggle to tell you what it was … might be able to broaden people’s understanding of what legislation is and where there may be areas that….could be changed.* ***(Male)***
Distinguishing affection from sexual intimacy	*it might be good to separate kissing and cuddling from sex … they are on different levels and we are social creatures, we are meant to be around other people and to interact physically, emotionally, mentally with others.* ***(Male)***
Problems with capacity and consent determination	*I’ve had a test of this recently with somebody living with dementia. And actually, they did deem her to have the capacity to consent. She does have dementia but does not have capacity around her own care needs, but she does have capacity to consent around sexual activity with a gentleman. But one of the hurdles she had to jump through was to talk about pregnancy and sexually transmitted diseases. And I just think that it actually, you know, if it’s a long-term relationship, do we need to do that with somebody when it’s not appropriate, pregnancy information is irrelevant at 80.* ***(Female)*** *Because the law does not make allowance for different types of expression of capacity, like through body language even if the individual cannot show that they have been able to go through the test of capacity. I think that different generations have different views on how capacity should be defined. I think that younger people like myself have different thoughts on this from the older generation.* ***(Male)***
Making it safe for everyone	*Even where sexual relation is allowed, it needs to be in the appropriate areas, because there may be other people in the vicinity that would find that inappropriate and upsetting and might find it terrifying, depending on how their views are on it. But I do agree with X, it is quite restrictive, but it’s sometimes trying to find that balancing act to enable somebody to, live an enriched life, but also protecting the individual and other people that could be around to make it safe for everyone.* ***(Male)***
Paid carers feel protective and err on the side of safeguarding	*The dangers are that we have vulnerable people and they could be abused and rape could occur. And obviously there is that tentative space in the middle. I agree that we need to protect the vulnerable people in society. It’s why we ended up with POVA [sic: Protection of Vulnerable Adults] originally. I remember when that came in, it was because all those things were occurring in the first place and POVA was to ensure that vulnerable adults are protected. But there is about protection and there is about right. So there has to be a balance, of course, because there is that risk area but there are rights too?* ***(Female)***
Voice of the populace influenced by ageism and cohort issues	*I spoke to a few people here about this issue over the last few days and you can see that people of my age who are sexually active and like talking about things like this are all up for allowing people with dementia to live life to the fullest whilst those who are not so open about this sort of thing are a little quiet about it.* ***(Male)*** *I think that different generations have different views on how capacity should be defined. I think that younger people like myself have different thoughts on this from the older generation.* ***(Male)***
Advance Decisions on Intimacy and other solutions	
Supporting intimacy and the assent model	*it should depend on the person as an individual, e.g., if they have been a highly sexualized person all their life, they’ve had a very active sexual life … it’s something that is meaningful to them and it brings them joy, pleasure so long as you know that it has already been pre-recorded as a well-known fact about the individual. And then, of course, the other person that’s involved, you know, it might even just be self-pleasure … that to me, should be fine. It’s just making sure you understand the background of that individual* ***(Female).*** *For me it all depends, if they are able show with their body language that they want the activity then they should be allowed. If they are not resisting or crying, or something like that, then they should be allowed as we are all social beings and we need to be able to interact that way with others … we have a lady here and she is not able to verbalise but you know that she knows what is happening around her. So that sort of person if they can show with their body language that they want intimacy then I see no reason why she should be stopped just because she cannot show that she has been able to go through the consent steps. You will know when she does not want you to wash her or help her in any way, she would kick and scream*. **(Male)**
Involvement of children as proxy sexual decision makers (and Power of Attorney) not the answer	*I don’t think that should be allowed. Because most parents choose their children as* Power of Attorney *and asking the children to make that sort of decision might end up affecting their children badly.****(Male)*** *The family might contradict any advance decisions because they may not have been involved when the decision was made … and try to fight that decision. The individual might already be in the advanced stages and then can’t be consulted about that. That would cause quite a lot of conflict … if an individual makes advance decision on intimacy they should communicate this to family members prior to them fully losing capacity so that they are aware that that’s actually what they want. But as an individual I don’t know if I would want my children involved if I make this decision.****(Female)***
Role of Court of Protection very limited	*[sic: Court of Protection] should only get involved on this issue to settle disputes between carers and the power of attorney. Court of Protection should also be able to revoke the advance directive if it becomes detrimental to the patient.* ***(Male)*** *I think that’s really a dangerous route to go down, for the sexual rights of individuals to be decided by courts rather than by the people who are around them and know them well. I think if we go down that legal front then, people who don’t know them well enough to make decisions will be getting involved in such decisions.* ***(Male)*** *it needs to be taken out of an emotive family situation, which is what* Power of Attorney *is and to be placed somewhere where it isn’t an emotive topic, where it can be judged on the person’s rights … that level Court of Protection would make sense because it’s removed from the emotions of family politics and relations.****(Female)***
Advance Decisions on Intimacy—*Pros—*gives voice to individual and specific sexual will and preferences, shared decisions between husband and wife	*Some people obviously just want to do it with a long-term partner, someone they might be married to for 60 years … .So, what’s right for one individual is not necessarily right for another individual. One individual might be able to write on that advance decision that if anyone shows them attention, they’re willing to kind of be part of a sexual activity. And someone might say, if my husband was still around, I would still like to consent to having sexual relationship with him. So, I do think that would be quite good, especially if it was written in depth rather than just I would you consent to sexual activity.* ***(Female)*** *I agree. I think it will be good if it is written out. For example, if an individual is married to, a husband, wife, whichever it may be, that they they’ve written down clearly that they would agree and they still want to maintain that intimacy with their husband or their wife. But should anything happen in the future, they still might find enjoyment from hand-holding. So maybe they would consent to hand-holding or, you know, just affection from another individual, but maybe not full sexual contact, they might be able to identify the areas that they’re happy with? And I think that’s an empowering thing because they’re still having that choice.* ***(Female)*** *everybody has a right to be loved and to be in love. …the way in which consent is right now needs to change … advance decisions would help.* ***(Female)*** *would be good if the people that would be involved in future sexual relations sit down and talk about what will be in the document. For example, husband and wife.* ***(Male)*** *[People Living With Dementia] can have their wants, needs and thoughts known and those wants, needs and thoughts can be taken into consideration and we can make them happen. So, to be able to carry on a loving relationship with a husband without that being questioned, as a vulnerable adult and not seen as rape as she can’t consent when her husband goes behind closed doors with her. These things are judged a lot you see.* ***(Female)***
Advance Decisions on Intimacy—*Cons—*change of mind	*So, there’s not really that understanding of what your life is going to be like in the future when this becomes valid. I’m not saying that it wouldn’t be without benefit, but I would treat it with caution because I think it will be difficult to sort of pre-imagine what that decision is going to be like when we lack capacity.* ***(Female)***
Advance Decisions on Intimacy—*Cons—*effect on partner or ending up in detrimental relationship	*The pitfalls might be the negative effects that it might have on the family and partner of the individual who is at home and does not have dementia. Say if the person with dementia starts off a relationship with someone else in the care home. Also, the advance decisions on intimacy might cause the person with dementia to end up in a relationship that might be detrimental to them.* ***(Male)***
Need for safeguards such as contemporaneous assessment on the day	*But for anything to happen, we need to make sure that all the safeguards for that person who lacks capacity are in place. and there’s the follow afterwards. For example, are they upset etc. It sounds all so clinical but we have to protect the person with dementia … I think someone will have to come out and meet with the couple privately. So, I think someone needs to come out, I don’t know who, and try to find out, what this person is like, what their dementia is like, how do they consent each day. They need to explore each case individually, almost like they do with the deprivation of liberty safeguards … they come out and do that. So why wouldn’t they come out for sexual relations?* ***(Female)*** *We want to make a decision on whether to allow this engagement to happen. And we got this prior information to say I really at this time would want this to happen. That’s going to go towards our decision-making process along with what the behaviour is on that day. So, in a way, you know, it’s leaning the carer toward supporting that decision, but the carer should maintain the right to* ***veto*** *If you think this just isn’t looking like what the individual wants on this day.* ***(Female)***

**Table 6.** Themes From Professionals With Expertise in the Care of Person Living With Dementia

Themes	Illustrative quote
Effects of thwarted sexuality on the person and their partner	*This could lead to the individual feeling confused about the “apparent sudden change” in what would have been a very normal and huge part of their relationship. For the partner it could lessen the bond between the couple* ***. (Female, Social Worker)*** *it further changes the dynamic in the relationship and could leave a wife of 60+ years feeling she isn’t even allowed to kiss and cuddle her husband for fear of it being considered wrong or bad.* ***(Female, Admiral Nurse)***
Discriminatory nature of the law	*It can prove discriminatory just because they have lost capacity to consent but still want to engage in intimate contact with their loved one.* ***(Female, Psycho-geriatrician)***
Adverse effect on well-being including link with changed behaviors	*For the person with dementia it might increase their frustrations and any agitation or aggression they may be experiencing as a symptom of their dementia.* ***(Female, Admiral Nurse)*** *in many cases of dementia, moments of lucidity where relationships with loved ones are remembered as they were. These lucid moments will of course include feelings of intimacy and if they are unable to express those emotions that can cause further deterioration in well-being.* ***(Female, Social Worker)***
Advance Decisions on Intimacy and other solutions	
Support for the assent model	*non-verbal communication and body language would be key to consideration around this … whilst the here and now consent is unobtainable I would want any care and support staff to be observing those non-verbal cues and relationships between myself and intimate partner to consider whether I am demonstrating consent.* ***(Male, Psycho-geriatrician)***
Better application of the concept of capacity and the threshold	*I think what the law could do better is how it applies the concept of capacity in this context. The bar/test for capacity in the circumstance I have described ought to be very low indeed.* ***(Female, Barrister)*** *Balancing the protection of vulnerable individuals with the recognition of their autonomy and rights is essential in any policy or guideline development process.* ***(Male, Dementia Ward Manager)***
Involvement of children as proxy sexual decision makers (and Power of Attorney) not the answer	*It may also be difficult for children or other family members of an individual with dementia, to consider that the individual is still expressing a desire for sexual intimacy when they do not necessarily express a desire for other forms of intimacy, which highlights the complexities of family dynamics and emotions when a loved one has dementia.* ***(Female, Social Worker)*** *Often the relevant person’s children hold* Power of Attorney*, they may not wish to be in a position to determine whether their [older adult] mother or father can consent to sexual relations. Equally, a spouse, may insist that the relevant person does engage in this, possibly compromising their safety.****(Female, Best Interests Assessor)***
Role of the Court of Protection limited	*A stranger would decide if you could partake in intimate acts—how would they know what was right for the person with dementia—a husband/wife would have to apply to the courts to be allowed to be intimate with the person they have been intimate with for 60+ years—it could be humiliating and maybe make them feel they are doing something wrong.* ***(Female, Admiral Nurse)***
Advance Decisions on Intimacy—*Pros—*gives voice, clarity for caregivers, facilitates intimacy and comfort	*provides clarity for caregivers, families are not left in a distressed state.* ***(Female, Psycho-geriatrician)*** *I advocate for any form of advanced care planning so that the individual’s voice is heard when they lack capacity in future. However, attitudes to sexual relations may legitimately change over time and also with the dementia affecting an individual’s thoughts and feelings and behaviour.* ***(Female, Social Worker)*** *It would give some legal protection to their loved ones and continue to make both parties feel that they remain in a marriage rather than carer/patient role—it gives the option back to someone with dementia otherwise strictly accordingly to law anyone with dementia would be devoid of all intimacy and perhaps for them (by extension) all source of comfort when we include cuddling, kissing and hand holding as intimacy. (* ***Female, Admiral Nurse)***
Advance Decisions on Intimacy—*Cons—*change of mind, risk of abuse by the partner	*attitudes to sexual relations may legitimately change over time and also with the dementia affecting an individual’s thoughts and feelings and behaviour.* ***(Female, Social Worker)*** *An Advanced Decision may imply consent to certain activities which an individual may no longer want to consent to when they see the world differently.* ***(Female, Social Worker)*** *For the less educated or more aggressive partners, the risk is that they would take the advanced decision literally and not pay attention to the “implied consent” and whether the person with dementia was happily going along with the act of intimacy or whether they were upset by the situation.* ***(Female, Admiral Nurse)***
Advance Decisions on Intimacy constrained by reliance on others for its enactment	*This sounds viable in theory. I wonder about the practical application. An advance decisions as to receiving or not receiving a form of treatment is workable because the maker of the advance decisions depends on a clinician or a team of them to deliver or not deliver that treatment; they can read and analyse the decision and act upon it. But a woman who makes an advance decisions that when dementia removes her decision-making capacity in relation to sexual activity enjoys less third-party involvement in the scrutiny of the way that advance decision is then used when she lacks decision making capacity. Her sexual partner is likely to be the person who “decides” that despite lacking capacity there and then to engage in sex, she nonetheless determined in her advance decisions that she would wish to have sex with that partner. But one does not choose to have sex with a partner in the future at all times and in all or any circumstances. There is the scope for quite a lot of decision making to be left to P’s partner as to whether it is her wish/feeling/will/preference to have sex in accordance with her generally granted advance decisions that she would like to do so. I fear that could cause problems for some people.* ***(Male, Barrister/Solicitor)***

### Overarching Themes: Commonalities and Distinctions

Commonalities across all stakeholder groups were (i) the law needs to change because of its hammer-like, blanket effect and failure to consider the myriad of individual circumstances and relationships, and human right violations; (ii) a dissonant theme of needing the law for protection; (iii) the adverse effects of thwarted sexuality has effects on both the individual and their partner; (iv) support for ADI with multiple caveats mandating checks and balances; (v) children are not appropriate proxy sexual decision makers; (vi) limited support for the role of the Court of Protection and Powers of Attorney in this space, with at best an adjunctive role policing operation of ADI; (vii) need to review the capacity concept and the threshold with support for an assent model.

The distinctive voice of people living with dementia was the sense of shame and stigma associated with this policing of their sexuality and the perception of being singled out for protection and intrusion into their lives. The distinctive voice of informal carers (wives of people living with dementia) was marked by themes of “what about me?” (reminding us that for the long-term partnered, this is a couple’s issue). Other themes from wives included protectiveness, but also concerns that ADI may fuel sexual obligation and powerlessness. The distinctive voice of people over 55 without dementia was marked by themes of fear of a future where their sexuality would be thwarted, the need to parcel out affection from intimacy, and a desire to make it easier for “those left behind “(family members) to know their wishes. The distinctive voice of paid carers and professionals was noted by their insight into the effects of the current laws on people living with dementia and their partners, as well as a need for a greater understanding of the law, and more guidance in the approach to capacity and consent in this space.

### Reflexivity

O. Sorinmade and C. Peisah are both old-age psychiatrists with extensive clinical and academic expertise in this area of sexuality in older persons and those living with dementia. A. Ruck Keene is a practicing English barrister and a Visiting Professor at King’s College London. Reflexive observation of the role of all three authors’ positions on ADI proponents was important in the research process. As such, we were careful to triangulate both our own perspectives and that of individual groups with those of other stakeholder groups as well as providing dissonant themes that highlighted the pitfalls of ADI. This reflexivity necessitated strong collaboration between coders, reliant on divergent opinions when it emerged. Further bias emanating from the third author’s (C. Peisah) feminist perspective and lived experience as both an unpaid carer, and professional with expertise in caring for people with dementia may have shaped data analysis. Finally, we were mindful of the need to find a balance between protection and empowerment. Ultimately, to respect the experience of all and to render it as a truly person-centered process, we need to equally support the needs of everyone from the sexually active to the sexually hyperactive and the sexually inactive.

## Discussion

Using the principle of deliberative democracy, this study qualitatively explores the views of a wide range of stakeholders regarding the expression of sexuality in persons living with dementia, the impact of the current laws (England and Wales) on such, and the need for law reform and the ADI proposition. As far as we are aware, this is the first study to seek to comprehensively capture stakeholder voice inclusive of persons living with dementia, paid and unpaid carers, persons over 55 without dementia, and healthcare professionals, regarding these issues. Commonalities in emergent themes across all stakeholder groups were (i) the law needs to change due to its hammer-like effect ignoring the individual and human right violations; (ii) a dissonant theme of needing the law for protection; (iii) the adverse effects of thwarted sexuality; (iv) support for ADI with multiple caveats; (v) less support for the role of the Court of Protection and Powers of Attorney; (vi) need to review the capacity concept and the threshold; and (vii) support for an assent model. The distinctive voices of people living with dementia, informal carers (wives of people living with dementia), and people over 55 without dementia were also noted.

The rich description of the importance of sexuality and affection in people’s lives, and the effects of this being thwarted voiced by all stakeholder groups, shows how integral these needs are to the well-being of persons living with dementia. At the same time, the need for safeguarding from abuse and exploitation was not forgotten. There was unanimous acknowledgment that getting this balance right of supporting enjoyment while also safeguarding is a morally, ethically, and legally complex issue with no easy answers.

Perhaps this complexity might be attenuated by parceling out “unnecessary problems” caused by professional and community ignorance and prejudice, from “necessary problems,” namely that the consent issue is simply very difficult legally. Based on our findings, there are things that look promising to try to deal with, both with regard to necessary and unnecessary problems.

Starting first with the easiest (by virtue of it being potentially amenable to education and training), the necessary problem of professional and community ignorance and prejudice. From the words of our stakeholders, there is a gaping lack of understanding of the law in this area among care staff. This overlaps with the related theme of problems with understanding consent and capacity in this area. We would hazard to guess that this knowledge gap extends beyond care staff to all professionals working with people living with dementia and their partners, given what we know about deficits in professional knowledge about capacity, consent and the law (Peisah, Lerman et al., 2021; Young et al., 2018). An important starting point is to improve knowledge about statutory (and human rights-compatible) presumption of capacity amongst all adults regardless of disability (Section 1(2), Mental Capacity Act, 2005). At present, to the contrary, there is an erroneous and human rights-incompatible “diagnosis-bound” presumption (O’Neill and Peisah, 2021) that the presence of dementia automatically means that a person lacks the capacity for sexual relations. Our data show the devastating effects of this overreach in protection. People living with dementia wonder why they warrant such intrusion and are left feeling ashamed of their sexuality, as if they “have been naughty” or “done something wrong.” When intimacy is thwarted, they can be left feeling unloved and abandoned.

Furthermore, given the strong support for the assent model and the lowering of threshold for consent articulated by our stakeholders, it is important to follow the clear judicial statements that it is necessary to focus on, but only on, the information that is actually relevant to the decision in question (*A Local Authority v JB* [2021] UKSC 52). As articulated by one informal carer “people might not understand the ins and out of the situation (sic: including ridiculously, pregnancy) as long as they understand the salient points.” This context also lends itself to support decision making which has both statutory and human rights-framework support (CRPD, 2016; House of Lords post-legislative scrutiny report, 2014). Education and training could target any of these unnecessary problems.

With regards to community attitudes, stakeholders identified the ageism-induced aversion to discussing sexuality among older people and the perception that older persons are asexual beings (D’cruz et al., 2020; Weeks, 2022), stifling both much-needed discussion and planning and facilitation of expression of sexuality. This is true both for the general community and the professional community in care homes, where resources such as the Sexuality Assessment Tool (Bauer, Fetherstonhaugh et al., 2014) can be utilized to help care facilities support the expression of sexuality of residents.

Regarding other necessary problems, many participants expressed the view that the relevant Criminal and Civil laws should be reviewed to accommodate the intimacy needs of people living with dementia without exposing them to harm and abuse. There was—perhaps understandably—no consensus as to how that should be achieved, but our study reinforces the fact that an opportunity was perhaps missed in 2000 to consult upon proposals advanced by the Law Commission of England and Wales to create provision in the criminal law for “apparently consensual sexual activity that takes place between two people, each with want of capacity, in non-exploitative circumstances; or where only one party to such activity lacks capacity, and this occurs in non-exploitative circumstances” (Great Britain Law Commission, 2000; Ruck Keene & Enefer, 2022)

A great richness of data regarding the pros and pitfalls of the ADI concept emerged. Although generally supportive, stakeholders identified the risks associated with the precedent self-binding authority with an instrument such as an ADI, fearing change of mind with the transformative nature of dementia (Hoffman, 2023).

What was novel and important was the understanding of relational autonomy (Kong, 2017; Walter & Ross, 2014) with consequences for both the person living with dementia and their partner, and the need to consider this as a couple’s decision. This was particularly salient for wives of this contemporary cohort of older persons (i.e., mean age of 72), who feared the potential effect of ADI on sexual obligation and coercion. Although other research has focused on comparative higher sexual obligation among older women in general (Lee et al., 2016; Nowakowski & Sumerau, 2019) and mixed findings regarding gendered differences in sexual obligation for persons with dementia (Lindau et al., 2018; Shen and Liu, 2023), the perspectives of female partners of persons living with dementia are important.

### Limitations

First, as we stated from the outset, this study was located in England and was specific to England, and thus specific to stakeholder response to laws as they operate in England and Wales. Having said this, the voices of stakeholders regarding the needs and rights of persons living with dementia and their partners have relevance globally. Second, the unsaturated nature of our perhaps most important groups, our lived experience groups of people living with dementia and unpaid, informal carers, was problematic. Perhaps the most glaring absence from our study is the voice and experience of LGBTQ+ people, rendering major limitations with regard to inclusion and diversity, particularly regarding gender and sexual orientation. Also, we did not obtain partnership status from participants living with dementia and three of four of our unpaid carers were spouses, our study thus failed to capture the voice of uncoupled persons. Explicit acknowledgment of potential variations in experiences among LGBTQ+ and noncoupled persons must be addressed in future research with more purposeful recruitment of this often-rendered invisible population (Peisah et al., 2018). Furthermore, details regarding dementia subtype and severity were neither collected for the participants living with dementia, nor in relation to the care recipients of the carer participants. This has an impact on the generalizability of our findings given the likely variability of sexual expression and needs across different dementia subtypes and stages. However, notwithstanding these omissions, we note that the human condition around sexuality is so diverse that it might not be possible to saturate themes for these groups. Third, what we gained from using the research question-guided Deliberative Democracy method, we may have lost by the directive, less exploratory nature of the interview. Notwithstanding this, a richness of data and “thick description” was still generated (Braun & Clarke, 2013). Finally, the unique gender-influenced view of the different needs of female partners emerged but needs further exploration as does the ADI concept.

### Implications for Practice

Over and above demonstrating a need for law reform, this snapshot of voices from a broad range of stakeholders, albeit limited, has a range of practical implications. First, the voices of protest regarding ageism and discrimination of people living with dementia, their carers, and people over 55 mandate public awareness-raising campaigns addressing ageism and ableism, focusing on society’s view that older people are invisible, unattractive, and asexual. Second, the voices of health professionals and care staff suggest a need for professional education regarding human rights principles of autonomy, dignity, and abuse safeguarding. Third, unanimous voice of protest regarding the common assumption that a diagnosis of dementia precludes the capacity to decide to engage in sexual relations, means we must always start first with a presumption of capacity as is reflected in English law, and we need consensus as to when, and how, capacity to decide to engage in sexual relations should be assessed (O’Neill & Peisah, 2021; Peisah, Ayalon et al., 2021). Fourth, care staff should be tasked with meeting needs for intimacy—when they exist—and facilitating, not thwarting sexuality (Bauer, Nay et al., 2014) for fear of sanction. Given the variation in human experience, part and parcel of this is understanding individual needs—or lack thereof—for intimacy and expression of sexuality to deliver truly person-centered care. Finally, it is high time that assessment of relational health is included in accreditation standards in care homes.

## Conclusion

We do not shy away from this issue because of its complexity. At stake are the well-being and human rights life of people living with dementia and their partners, and their ability to live well with dementia both in the community and in care settings. Although our aspirations are modest, at minimum fostering awareness raising and discussion around dementia, sexuality, and the law; in this area where there is so much more to be done, discussion per se has its own inherent value (Meeks, 2023).

## Supplementary Material

gnae112_suppl_Supplementary_Material

## Data Availability

The anonymized interview manuscripts and analytic methods can be made available on request. Pre-registration of the study was not mandatory. This research was approved by the Wales REC 2.
