# Supplementary material for: The Law, Dementia, and Sexuality—Is the Law Striking the Right Balance?
Source: Gerontologist. 2024 Aug 14;64(11):gnae112. doi: 10.1093/geront/gnae112 (PMC11472172; doi:10.1093/geront/gnae112)
Supplement: gnae112_suppl_Supplementary_Material [file gnae112_suppl_supplementary_material.docx]

**Supplementary Material**

**Supplementary File 1**

**Interview Schedules**

1. **Interview Schedule for People Living with Dementia**
   **Prompt 1** 
   Do you think that it is a problem when people living with dementia are dissuaded from engaging in sexual relations because they can no longer consent?

   (a) if yes, why?  

   (b) something that we should look into and if yes, 

   (c) how do you think that we should look into this issue?  


   **Prompt 2:** 

   Please tell us if you think that people who have dementia regardless of severity, should continue having sexual relations, as long as it is safe for them to do so, even if they have lost the ability to give permission for such activities?   - *Explore Yes or No answer.*

**Prompt 3:** 

Do you have any suggestions on possible amendments/changes to the law as it relates to people with dementia in the area of sexual relations even in the moderate to severe stages of the illness? Do you think that the law needs to be looked at? ----- pls tell us some more.

1. **Interview Schedule Carers**

We would appreciate it if you kindly help answer the questions below:

(a). Do you think that an individual with dementia who cannot consent to sexual relations should be dissuaded from engaging in such activity?

b). Secondly, do you think that research should be done on the subject of sexual relations in people living with dementia to explore how the present laws impact people living with dementia and their loved ones?

(c) Thirdly, we are running focus groups, but do you think that there are other ways by which we should study this topic - for example sending out questionnaires by post to people and so on and so forth?

Prompt 1

Do you think that the law as it is now, is striking the right balance when it comes to sexual relations in People Living With Dementia especially individuals who have lost the capacity to engage in such relations?

Follow up questions.

. Do you think that the law should change as it relates to people living with dementia and their engagement in sexual relations, especially after they lose the ability to consent to such relations?

Prompt 2:

One suggestion that has been made about how the law could *change (to protect the* People Living With Dementia *from exploitation and abuse and to protect the other person that they engage in sexual relations with),* would be to allow people to say in advance what they would or would not consent to if they lost the ability to give ‘here and now’ consent in future- a sort of ADVANCE DECISION ON INTIMACY - What are your thoughts about this suggestion?

Prompt 3:

Another suggestion that has been made about how the law could change would be to allow for holders of Health and Welfare Power of Attorney or for the Court of Protection to have the authority to decide on whether an individual, who has lost the ability to consent to sexual relations, should engage in such relations.

What are your thoughts about this suggestion?

Prompt 4:

If the Law does change to allow people to say in advance what they would or would not consent to, what are your thoughts about the choices that an individual should be able to make about future sexual relations - limited (just cuddling, intimate hand holding, kissing) or unlimited choices?

Prompt 5:

If the Law does change to allow holders of Health and Welfare Lasting Power of Attorney or the Court of Protection to make decisions about sexual relations on behalf of people with dementia who can no longer make such decisions by themselves, what are your thoughts about the choices that HWLPA or Court of Protection should be able to make in such situations- should their decisions be limited to allowing the individual to cuddle, kiss etc or should they be able to make any decisions regarding sexual relations in the individual with dementia.

Prompt 6

What, if anything, would you like to see happen to the law or policy guidelines in relation to People Living With Dementia with regard to their engagement in sexual relations

1. **Interview Schedule People over 55 without Dementia**

We would appreciate it if you kindly help answer the questions below:

(a). Do you think that an individual with dementia who cannot consent to sexual relations should be dissuaded from engaging in such activity?

b). Secondly, do you think that research should be done on the subject of sexual relations in people living with dementia to explore how the present laws impact people living with dementia and their loved ones?

(c) Thirdly, we are running focus groups, but do you think that there are other ways by which we should study this topic - for example sending out questionnaires by post to people and so on and so forth?

Prompt 1:

Do you think that the law as it is now, is striking the right balance when it comes to sexual relations in People Living With Dementia especially individuals who have lost the capacity to engage in such relations?

Do you think that the law should change as it relates to people living with dementia and their engagement in sexual relations, especially after they lose the ability to consent to such relations?

How would you define consent, and why would you say that consenting to sexual relations is important?

**Statement**

- Dementia can affect anyone, and we want to further explore some issues with you.
- Imagine the hypothetical situation that you develop dementia and become unable to give “here and now” consent to sexual relations:

Prompt 2:

- How do you think that you might be affected in that hypothetical situation if you are prevented from engaging in sexual relations?
- If you could do anything now, to mitigate that future hypothetical situation, what might you do?

Prompt 3:

One suggestion that has been made about how the law could *change (to protect the People Living With Dementia from exploitation and abuse and to protect the other person that they engage in sexual relations with),* would be to allow people to say in advance what they would or would not consent to if they lost the ability to give ‘here and now’ consent in future- a sort of ADVANCE DECISION ON INTIMACY - What are your thoughts about this suggestion?

Prompt 4:

Another suggestion that has been made about how the law could change would be to allow for holders of Health and Welfare Power of Attorney or for the Court of Protection to have the authority to decide on whether an individual, who has lost the ability to consent to sexual relations, should engage in such relations.

What are your thoughts about this suggestion?

Prompt 5:

If the Law does change to allow people to say in advance what they would or would not consent to, what are your thoughts about the choices that an individual should be able to make about future sexual relations - limited (just cuddling, intimate hand holding, kissing) or unlimited choices?

Prompt 6:

If the Law does change to allow holders of Health and Welfare Lasting Power of Attorney or the Court of Protection to make decisions about sexual relations on behalf of people with dementia who can no longer make such decisions by themselves, what are your thoughts about the choices that the Health and Welfare Lasting Power of Attorney or Court of Protection should be able to make in such situations- should their decisions be limited to allowing the individual to cuddle, kiss etc or should they be able to make any decisions regarding sexual relations in the individual with dementia.

Prompt 7:

What, if anything, would you like to see happen to the law or policy guidelines in relation to People Living With Dementia with regard to their engagement in sexual relations?

1. **Survey questionnaire for Professionals with expertise in the care of People Living With Dementia**

**a.** In the case of an individual living with dementia, who has lost the "here and now" ability to consent to sexual relations, the position of the law, is that they should no longer engage in sexual relations. What effects might stopping them from engaging in sexual relations have on them and their loved ones?

**b.** Please comment on whether the law as it is now, is striking the right balance between protecting people living with dementia, who have lost the capacity to consent to engage in such relations, and enabling them to continue engaging in sexual relations as part of basic human activities?

**c.** It is lawful under Section 24 of the Mental Capacity Act 2005, to make Advance Decision to Refuse Treatment. *There is the suggestion for the law to change, to make it lawful to make Advance Decision on Intimacy.*

*The Advance Decision on Intimacy would allow people to say in advance how they would like to be treated regarding sexual relations, if they lose the ability to give ‘here and now’ consent to sexual relations in future.*

- What are your thoughts about the pros and cons of this suggestion?

**d.** If making advance decision on intimacy is lawful, would you make one yourself? Please comment.

**e.** There is the suggestion for the law to change to amend s.9 MCA 2005 to empower Health and Welfare Attorneys to be able to decide on whether an individual who has lost the capacity to engage in sexual relations should engage in such relations or not.

- What are your thoughts about the pros and cons of this suggestion?

**f.** Another suggestion is for the law to change to amend s.27 MCA 2005 to empower the Court of Protection to be able to decide on whether an individual who has lost the capacity to engage in sexual relations should engage in such relations or not.

- What are your thoughts about the pros and cons of this suggestion?

**g.** What might you like to change, if possible, in policy and guidelines, to improve the welfare of People Living with Dementia?
